# Successful Regenerative Endodontic Therapy of a Dens Evaginatus Mandibular Second Premolar with an Acute Apical Abscess and Extensive Periapical Bone Loss: A Case Report

**DOI:** 10.1055/s-0044-1791218

**Published:** 2024-10-11

**Authors:** Francesco J. DeMayo, Jackson T. Seagroves, Takashi Komabayashi

**Affiliations:** 1 Division of Comprehensive Oral Health – Endodontics, Adams School of Dentistry, University of North Carolina at Chapel Hill, Chapel Hill, North Carolina, United States

**Keywords:** acute apical abscess, necrotic pulp, permanent, regenerative endodontics

## Abstract

Regenerative endodontics is a rapidly growing field within dentistry that aims to replace damaged tooth structures and cells of the pulp–dentin complex. This case report presents the successful management of an immature permanent second mandibular premolar with an acute apical abscess and extensive apical bone loss caused by a dens evaginatus. This tooth was unexpectedly treated with long-term calcium hydroxide (Ca(OH) _2_ ) that was replenished several times over 6 months. During the treatment process, this tooth became reinflamed developing a chronic apical abscess after resolution of the initial acute apical abscess, which resulted in the replacement and prolonged use of Ca(OH) _2_ . The final regenerative procedures were completed using a bioceramic fast set putty placed directly over the blood clot and an occlusal composite restoration. At the 6-month follow-up, the patient was asymptomatic with a fully healed radiographic lesion, new periodontal ligament, and the apex closed with thickening of the mesial and distal aspects of the root. At the 15-month follow-up, the patient remained asymptomatic with continued evidence of radiographic development of the apical aspect of this tooth, displaying root end closure and thickening. This is a unique case report of the management of a complex infection process in an immature tooth with regenerative techniques with repeated and long-term use of Ca(OH) _2_ . This novel report provides dental practitioners with a new potential protocol for the management of these immature cases with advanced periapical infections that require extensive disinfection to have successful outcomes.

## Introduction

Regenerative endodontics is a concept that has been present in the endodontic discipline since the 1970s. 1 According to the American Association of Endodontists' Glossary of Endodontic Terms in 2020, regenerative endodontic treatment aims to act as biologically based procedures designed to physiologically replace damaged tooth structures, including dentin and root structures, as well as cells of the pulp–dentin complex. 2 The three general requirements for this tissue engineering procedure in regenerative endodontics are stem cells, growth factors, and some form of scaffold. 3 This form of treatment also has three goals: (1) eliminating symptoms and having evidence of bony healing, (2) increasing root wall thickness and root length, and (3) a positive response to sensibility testing. 4 However, interest in regeneration in endodontics did not gain momentum until 2001 when a Japanese case report using double antibiotic paste (metronidazole and ciprofloxacin) was able to display successful treatment of a permanent mandibular second premolar with a sinus tract in a 13-year-old patient. 5 These results were replicated several years later in 2004 in an American case report that used a triple antibiotic paste (metronidazole, ciprofloxacin, and minocycline) 6 in a permanent mandibular second premolar with a sinus tract in an 11-year-old patient. 7

The popularity of this field has grown significantly since these case reports. Some authors have called for regenerative endodontics to be considered the first line of treatment for immature teeth with pulpal necrosis and apical periodontitis. 8 Even dentists have become more accepting of these procedures and are searching for additional training in the field. 9 As the field begins to advance, it is important to document the treatment of different clinical scenarios that can arise in practice to serve patients better and improve treatment outcomes. Especially, common dental pathologies such as this one in mandibular premolars are caused by a dens evaginatus. Dens evaginatus is believed to be caused by the evagination of the internal enamel epithelium and dental papilla into the stellate reticulum during morphodifferentiation. 10 This results in the formation of a tubercule on the occlusal surfaces, commonly on mandibular premolars, due to occlusal trauma can fracture away, producing a pathway for bacteria to the pulp that causes necrosis and periapical pathology. 10 11 Therefore, dentists must have a solid protocol to deal with cases like these since they will certainly present themselves in practice. This case report aims to provide the successful management of a necrotic mandibular second premolar presenting with an acute apical abscess caused by dens evaginatus in a 10-year-old patient utilizing EndoSequence BC RRM Fast Set Putty (Brassler, Savannah, Georgia, United States).

## Case Report

A 10-year-old male patient initially presented to his referring dentist on May 13, 2022, with swelling as an emergency evaluation. This patient was ultimately prescribed a 7-day course of amoxicillin–clavulanate (Augmentin) 875 to 125 mg by the referring dentist and referred to the Graduate Endodontic Clinic at the University of North Carolina at Chapel Hill (UNC-CH) Adams School of Dentistry (ASOD). This patient presented with his mother to the Graduate Endodontics Clinic, UNC-CH ASOD to be evaluated by the first provider for a dental infection in the mandibular right quadrant causing swelling and pain on May 26, 2022. As a result, since the patient had completed his original antibiotic prescription, his swelling had subsided by this appointment. The patient's chief complaint was “My tooth has been starting to give me trouble, and my dentist says I need a root canal.” The patient's medical history consisted of previous atopic dermatitis, previous parotitis, positive antinuclear antibodies, high pediatric body mass index more than 95% for his age, and enuresis. The patient reported no history of drug allergies, current medications, or cardiac/joint disease requiring antibiotic prophylaxis. Based on this medical presentation, the patient was designated American Society of Anesthesiologists (ASA) physical status Class II.

**Fig. 1:**
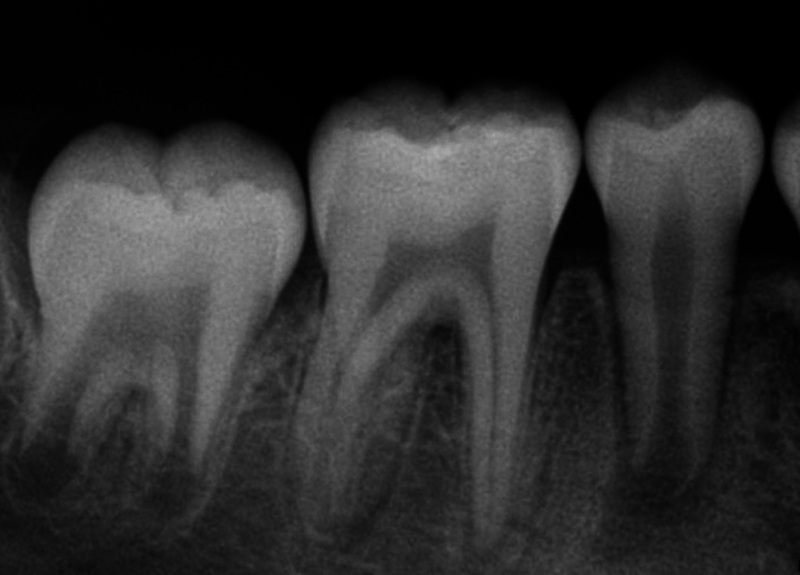
Preoperative periapical radiograph of tooth #29 taken from referring provider.

The patient's dental history included routine dental care, moderate caries risk, and relatively good oral hygiene. The extraoral examination and perioral soft tissue examination were within normal limits. His temporomandibular joint function was normal, without any deviation upon opening or discomfort upon palpation. The intraoral examination at this appointment revealed no soft tissue swelling, sinus tract, or isolated deep probing depths around teeth #27 (right mandibular canine) through #30 (right mandibular first molar). Tooth #30 presented with an occlusal composite restoration with intact margins. The dental provider at this appointment determined there was no percussion (vertical and horizontal) or palpation sensitivity of teeth #27 through #30. Electric pulp testing was not utilized at this appointment, and cold testing revealed a negative response to tooth #29 (right mandibular second premolar). Radiographically, this provider noted immature apices and incomplete root formation of teeth #28 (right mandibular first premolar) and #29 with high pulp horns present ( Figs. 1 2 3 4 ). Tooth #29 was diagnosed as pulpal necrosis with asymptomatic apical periodontitis and was scheduled for treatment.

**Fig. 2:**
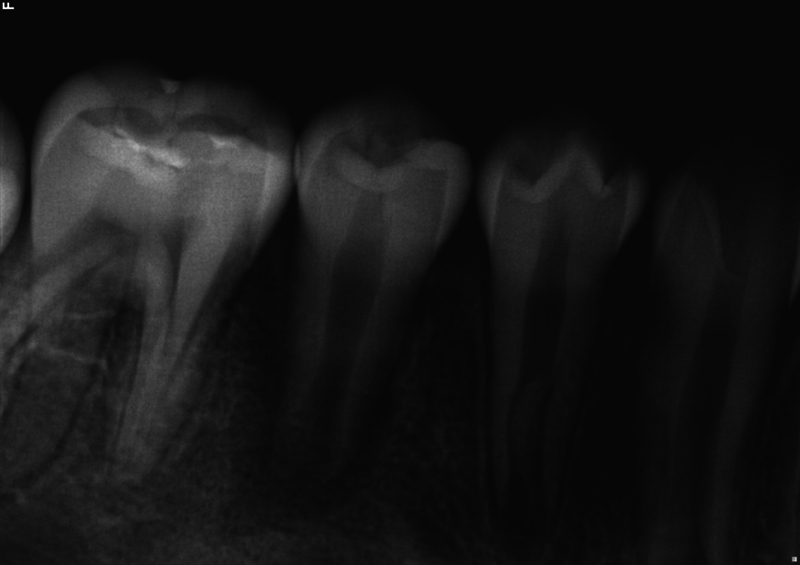
Preoperative periapical radiograph of tooth #29 taken at the University of North Carolina at Chapel Hill Adams School of Dentistry.

**Fig. 3:**
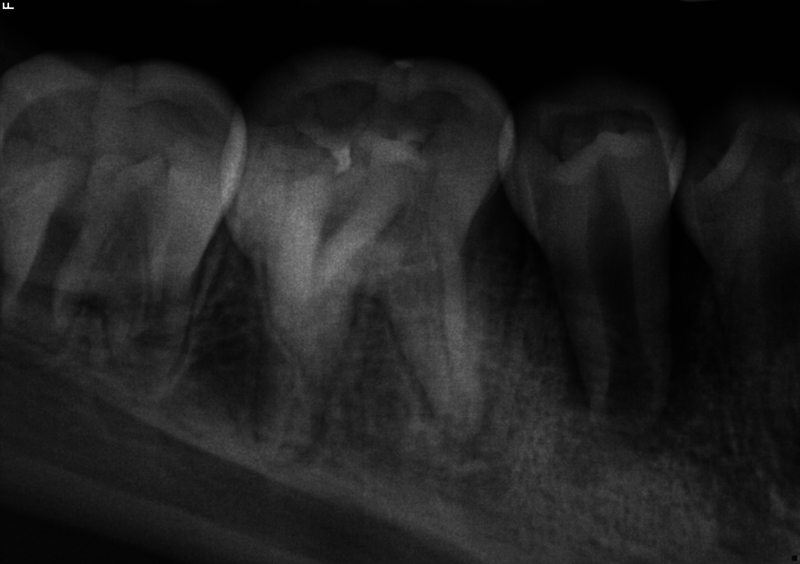
Preoperative periapical radiograph of tooth #29 distally shifted taken at the University of North Carolina at Chapel Hill Adams School of Dentistry.

**Fig. 4:**
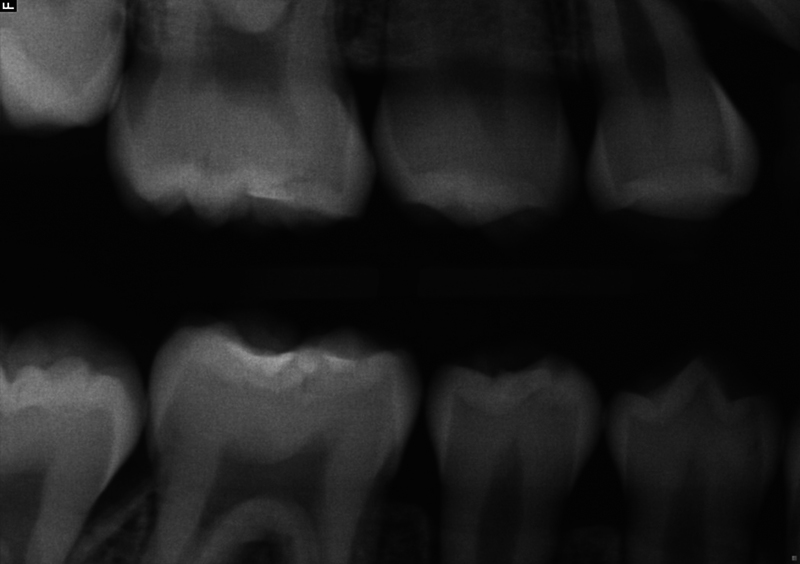
Preoperative bitewing radiograph of tooth #29 taken at the University of North Carolina at Chapel Hill Adams School of Dentistry.

The patient and mother returned to Graduate Endodontic Clinic, UNC-CH ASOD on June 13, 2022, and saw another provider who would ultimately provide all treatment. At this appointment, the patient presented with buccal swelling in the right mandibular quadrant and a chief complaint of “I have swelling on my gum.” However, the patient denied symptoms of pain. While an extraoral examination revealed no facial swelling, facial asymmetry, or swollen lymph nodes present, an intraoral examination showed a buccal swelling was present in the proximity of tooth #29 with deep probing depths along the buccal of tooth #29. The area around tooth #29 was palpation tender; however, tooth #29 was not percussion (vertical and horizontal) sensitive and had no mobility. Cold testing was within normal limits for teeth #30, #28, and #27 but was negative for tooth #29. Cold testing was repeated several times per tooth to confirm the results. Electrical pulp testing was not performed at this appointment. An exposed remnant of a dens evaginatus tubercle was noted on the occlusal of tooth #29. Radiographically, a large periapical radiolucent lesion (∼7 mm in diameter and extending up the root mesially and distally to the marginal bone level [ Fig. 2 ]) was present at the apex of tooth #29 with a dens evaginatus that had already fractured prior to this appointment ( Figs. 1 2 3 4 ). This periapical lesion displayed extensive bone loss that extended along the mesial and distal aspects of the roots ( Figs. 1 2 3 ). Teeth #27, #28, and #30 were diagnosed as normal pulpal tissue with normal periapical tissue, and tooth #29 was diagnosed with pulpal necrosis with an acute apical abscess. These diagnoses were formulated based on the sensibility testing, with tooth #29 being the only tooth that did not respond to cold after several attempts, clinical examination with swelling within the vicinity, and radiographic examination displaying a periapical radiolucency at the apex of this tooth with a dens evaginatus coronally providing an avenue for bacteria to reach the pulp chamber.

The patient was provided with treatment options for nonsurgical root canal treatment with apexification or apical plug placement, extraction with/without replacement options, or regenerative endodontic treatment. The patient- and mother-decided regenerative treatment was deemed acceptable because of the patient's age and immature apex/incomplete root development with an initial radiographic estimated apical opening of 1.4 mm, estimated mesial root thickness of 0.7 mm, and estimated distal root thickness of 1.1 mm in the apical third of tooth #29. The patient and his mother completed the treatment consent process.

**Fig. 5:**
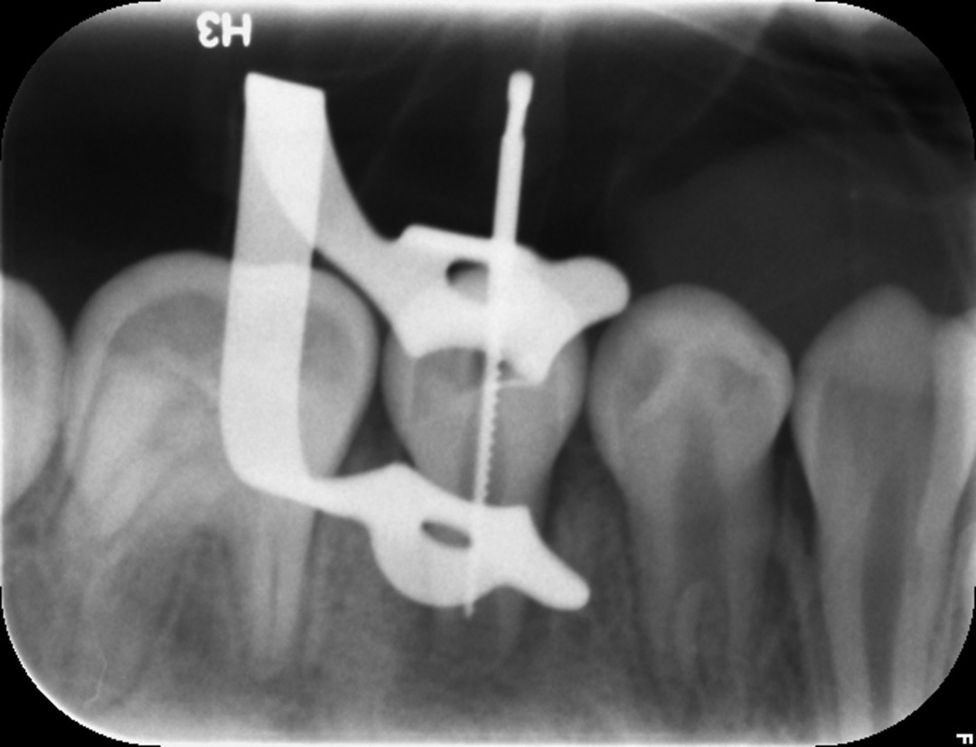
Working length periapical film with #60 K file.

Treatment was initiated at this appointment to alleviate the patient's symptoms and swelling. The patient was anesthetized with 1.7 mL of 4% articaine with 1:100,000 epinephrine (Septocaine; Septodont, New Castle, Delaware, United States) by buccal and lingual infiltration around tooth #29. Single tooth isolation was achieved for tooth #29 with a dental clamp and rubber dam. Access was achieved with a number 4 round carbide bur on a high-speed handpiece with copious amounts of water coolant removing the occlusal remnants of the dens evaginatus, entirely leading to the pulp chamber. Upon access to the pulp chamber, a single canal was located, and the presence of necrotic pulpal tissue was confirmed. Restorability was established, and working length was determined using an electronic apex locator (Root ZX II, J. MORITA MFG. CORP., Kyoto, Japan); measurement film was taken with a 60 K file (Dentsply-Sirona, Johnson City, Tennessee, United States) ( Fig. 5 ).

The working length was adjusted accordingly and determined to be 18 mm, with the buccal cusp serving as the landmark for the rubber stopper. The single canal was instrumented with nickel–titanium hand files to a size 80 with a 4% taper (Dentsply-Sirona). During the cleaning and shaping, copious irrigation was used between files with 4% sodium hypochlorite (NaOCl) (Henry Schein, Melville, New York, United States). A final rinse of 5 mL of 17% ethylenediaminetetraacetic acid (EDTA) (Henry Schein) was used to remove the smear layer, followed again by 10 mL of 4% NaOCl solution. EndoActivator (Dentsply-Sirona) activated the final rinse solutions for three 10-second intervals for each solution. The canal was dried with large-size endodontic paper points (Dentsply-Sirona); however, it was difficult to dry the canals completely at this appointment due to the extent of the bacterial infection. UltraCal XS Ca(OH) _2_ paste (Ultradent, South Jordan, Utah, United States) was placed as an intracanal medicament, and a sterile sponge was placed in the access. As a temporary material, the access was sealed with 3 mm of GC Fuji Triage (GC America, Alsip, Illinois, United States).

After temporization, an incision and drainage procedure was performed with a 15C blade placed through the center of the swelling to the osseous structures on the buccal of tooth #29. A hemostat was used to break up the tissue, and purulent drainage was achieved. Copious irrigation with sterile saline solution was used, and the lesion was digitally compressed to achieve more drainage. The incision was left open to allow for more drainage. The patient was discharged from the clinic chair and is in good standing.

**Fig. 6:**
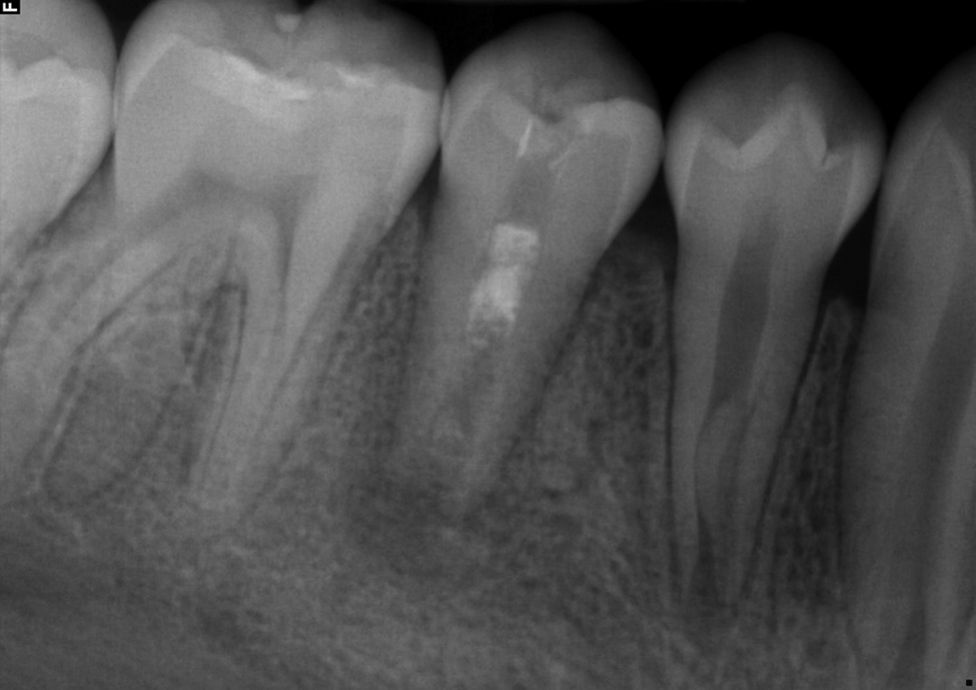
Urgent care periapical film of partially washed-out calcium hydroxide medicament.

The patient and mother returned to UNC-CH ASOD for a third appointment with a third provider in the Graduate Endodontic Clinic on October 17, 2022, as an urgent care setting. The patient reported occasional pain when biting on tooth #29 and the presence of a sinus tract. A periapical radiograph was exposed that revealed the Ca(OH) _2_ had partially washed away ( Fig. 6 ). The provider retested teeth #28 and #30, which revealed cold testing within normal limits, and no gross caries were present on these teeth. Tooth #29 had an intact provisional access restoration, such as GC Fuji Triage, on the occlusal surface. The provider at this appointment recommended that the patient return for further disinfection and the placement of a second round of Ca(OH) _2_ as an intracanal medicament with the original treating provider.

**Fig. 7:**
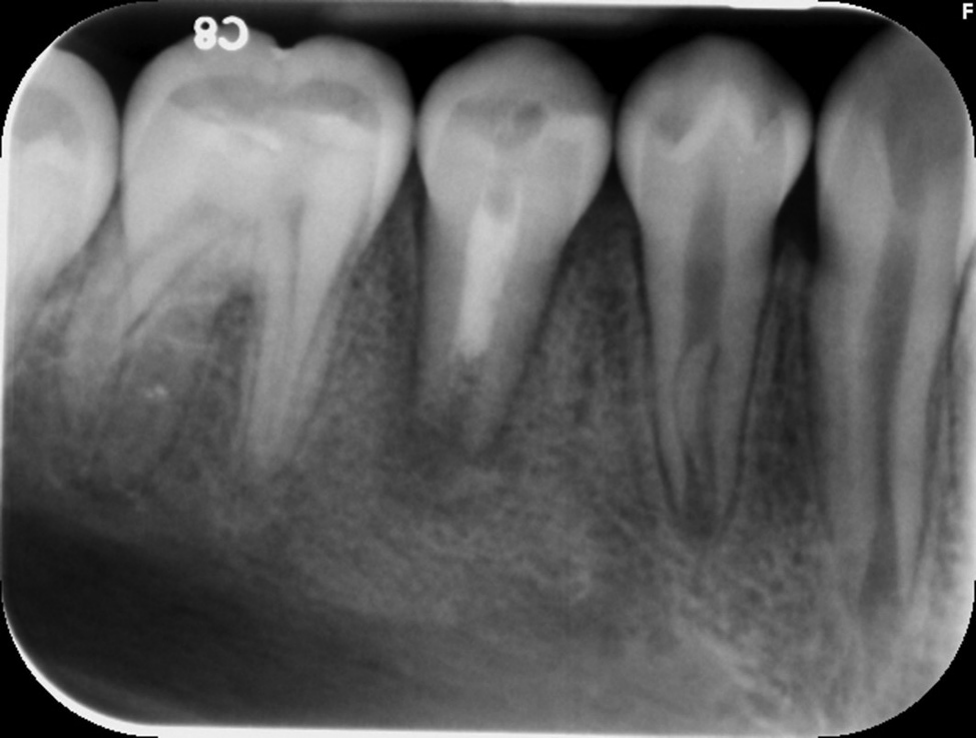
Postoperative calcium hydroxide placement second treatment appointment.

The patient presented with his mother for a second treatment appointment the next day. The sinus tract was still present. The same technique as the first treatment appointment was used for local anesthesia and rubber dam placement. A single canal was again accessed, removing the provisional access restoration and existing Ca(OH) _2_ . It was instrumented with nickel–titanium hand files to a size 100 with a 4% taper (Dentsply-Sirona). During the cleaning and shaping, copious irrigation was used between files with 4% NaOCl (Henry Schein). A final rinse of 5 mL of 17% EDTA (Henry Schein) was used to remove the smear layer, followed by 10 mL of 4% NaOCl solution. The final rinse solutions were activated by gutta-percha cone pumping for three 10-second intervals for each solution. The canal was dried with large endodontic paper points (Dentsply-Sirona). UltraCal XS Ca(OH) _2_ paste (Ultradent) was placed as an intracanal medicament, and a sterile sponge was placed in the access. As a temporary material, the access was sealed with 3 mm of GC Fuji Triage (GC America) ( Fig. 7 ).

**Fig. 8:**
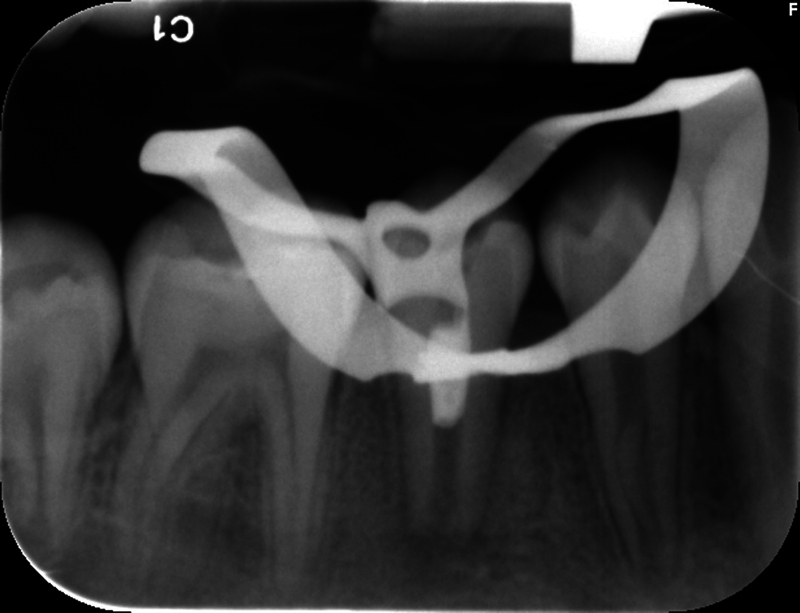
Obturation periapical radiograph with EndoSequence BC RRM Fast Set Putty.

**Fig. 9:**
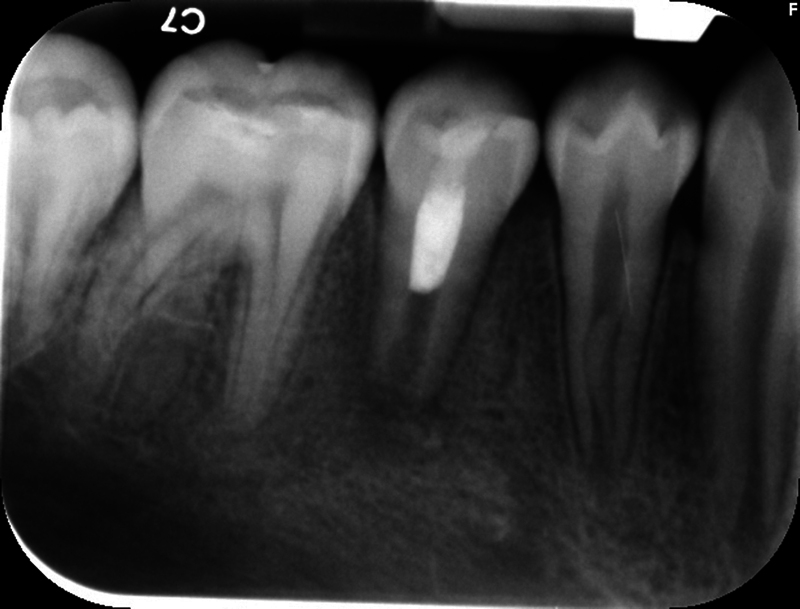
Immediate postoperative periapical radiograph of tooth #29.

The patient returned approximately 2 months later, on December 5, 2022, for the final treatment appointment. The sinus tract had healed upon clinical examination, and the patient denied symptoms. Therefore, it was determined that the infection was now under control. The patient was provided buccal and lingual infiltration with 3.4 mL of 3% mepivacaine with no epinephrine (Dentsply-Sirona). The same procedure was initiated for rubber dam isolation. The single canal was again accessed by removing the existing Ca(OH) _2_ with 10 mL of sterile saline solution; 10 mL of 17% EDTA (Henry Schein) was irrigated. Next, a precurved 15 K file was used to instrument 2 mm past the apex (20 mm) to help stimulate bleeding into the canal. The blood clot, which provides scaffold, growth factors, and stem cells, came to approximately mid-root. Approximately 5 mm of EndoSequence BC RRM Fast Set Putty (Brassler) was then placed directly over the blood clot to the level of the cementoenamel junction ( Fig. 8 ). The material was placed directly over the blood clot because it does not begin the setting process until it is in contact with a moist environment. 12 The material was allowed to be set for 20 minutes until hardened based on the manufacturer's recommendations. 12 The access cavity was then filled with a permanent coronal restoration shade A2 Filtek Supreme flowable composite (3M Oral Care, St. Paul, Minnesota, United States). Occlusion was checked with articulating paper and adjusted with a football-shaped carbide bur until lite contacts were present. A postoperative radiograph was exposed ( Fig. 9 ). Postoperative instructions were provided to the patient and mother, and the patient was discharged from the clinic chair.

**Fig. 10:**
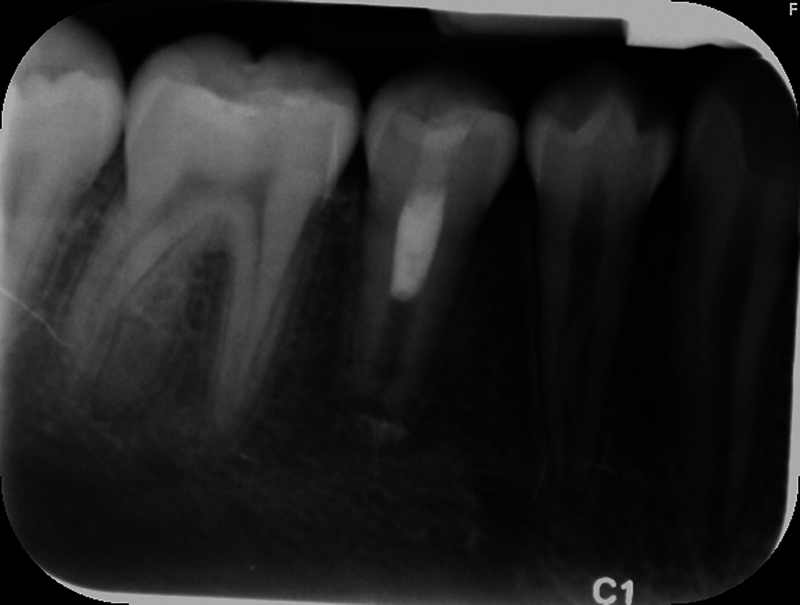
Six-month follow-up periapical radiograph.

The patient and mother returned for a 6-month follow-up appointment. The patient had experienced no symptoms since the completion of treatment, including pain in the hot/cold, pain in chewing, spontaneous pain, swelling, and sinus tract. An extraoral examination revealed no significant findings, and an intraoral examination of tooth #29 revealed no signs of swelling or sinus tract, mobility, isolated deep probing depths, percussion (vertical or horizontal), or palpation sensitivity. Tooth #29 did not respond to cold testing, and electrical pulp testing was not performed. The composite restoration appeared to remain intact. Radiographically, it appeared the periapical lesion had completely healed with the return of the entire periodontal ligament around the apical aspect of the root ( Fig. 10 ). Furthermore, the apical end of tooth #29 appeared to begin to close with dentinal thickening ( Fig. 10 ). However, the root did not appear to have increased in length or only minimally increased by 1 mm to an estimated radiographic length of 19 mm ( Fig. 10 ). Radiographic estimated mesial root canal wall of 1.6 mm and distal root canal wall thickness of 1.6 mm in the apical third of tooth #29 were noted ( Fig. 10 ). These measurements were made from the outermost radiographic aspect of the dentinal segment in the apical third of the root to the innermost aspect of the dentinal segment at that same location of the root for both the mesial and distal aspects of the root. The bioceramic material remained unchanged and still in place ( Fig. 10 ).

**Fig. 11:**
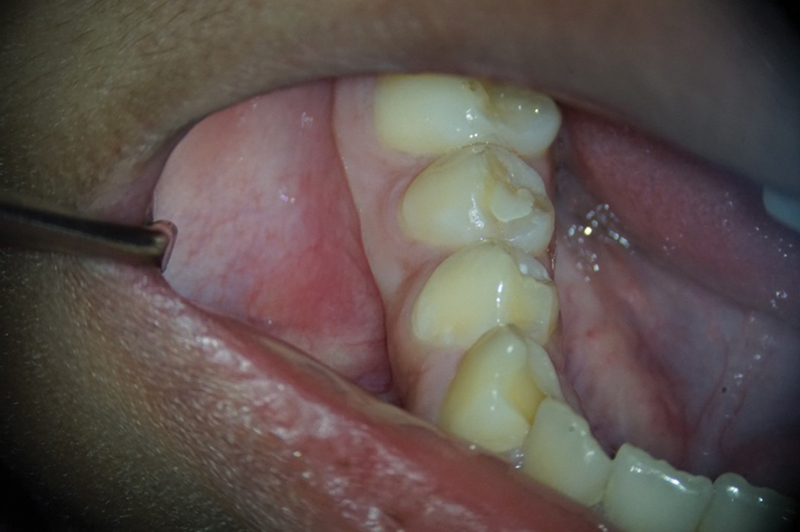
Intraoral picture of tooth #29 at 1-year follow-up. Permanent coronal restoration shade A2 Filtek Supreme flowable composite with intact margins.

**Fig. 12:**
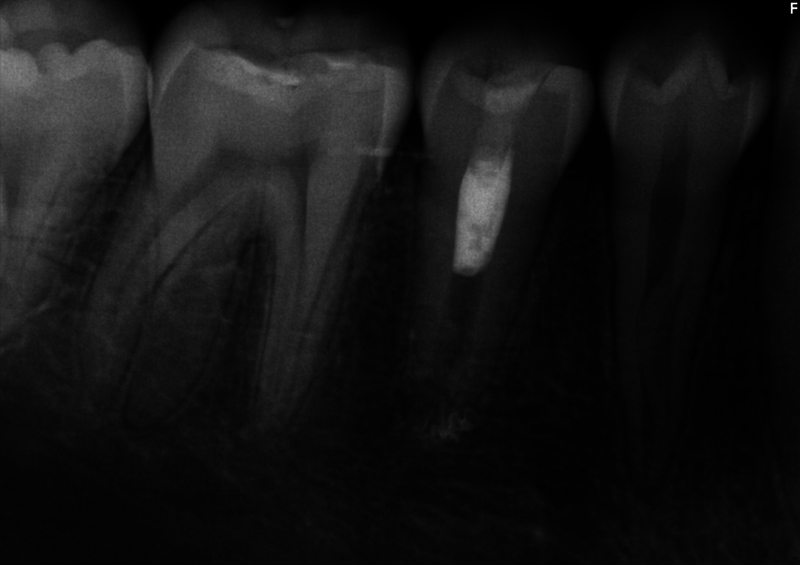
One-year follow-up periapical radiograph.

The patient returned for a 1-year follow-up appointment. The patient again denied symptoms since the 6-month follow-up appointment, including sensitivity to hot/cold, pain on chewing, spontaneous pain, swelling, and sinus tract. An extraoral examination revealed no significant findings. An intraoral examination of tooth #29 revealed no signs of swelling or sinus tract, mobility, isolated deep probing depths, percussion (vertical or horizontal), or palpation sensitivity ( Fig. 11 ). The occlusal composite restoration had intact margins with no signs of recurrent carious lesions and no crown discoloration ( Fig. 11 ). At this appointment, both cold and electrical pulp testing produced negative responses. Radiographically, the apical end of tooth #29 appeared to close further with dentinal thickening. Yet, the root did not appear to have increased in length, remaining at an approximate radiographic length of 19 mm ( Fig. 12 ). Approximately, the radiographic mesial canal wall in the apical third was 1.9 mm, and the distal canal wall in the apical third was 1.8 mm. These measurements were again made from the same outermost radiographic aspect of the dentinal segment in the apical third of the root to the innermost aspect of the dentinal segment at that same location of the root for both the mesial and distal aspects of the root.

**Fig. 13:**
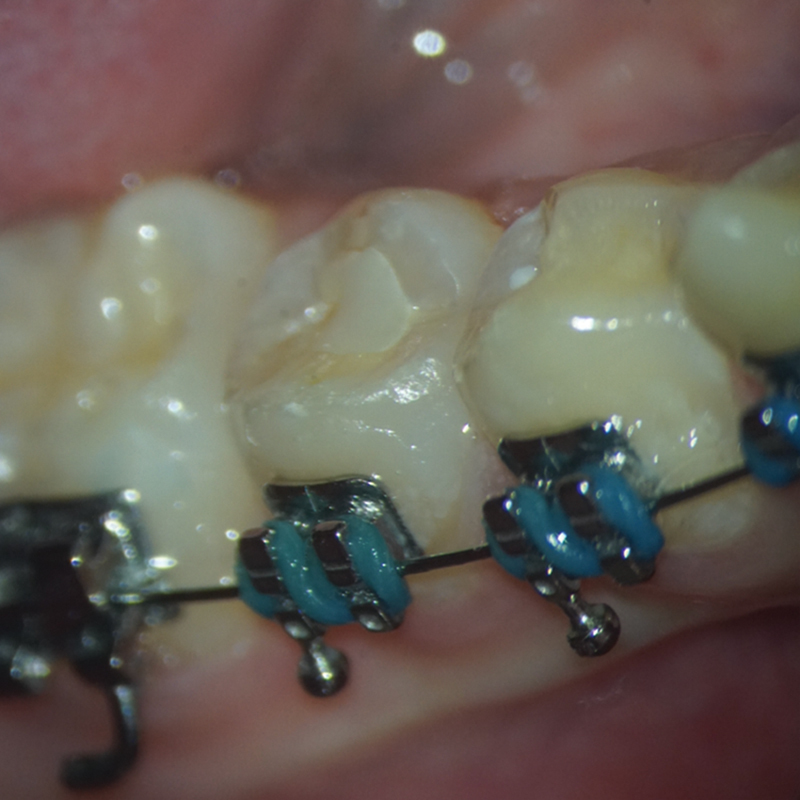
Intraoral picture of tooth #29 at 15-month follow-up. Permanent coronal restoration shade A2 Filtek Supreme flowable composite with intact margins and orthodontic wire and brackets present.

**Fig. 14:**
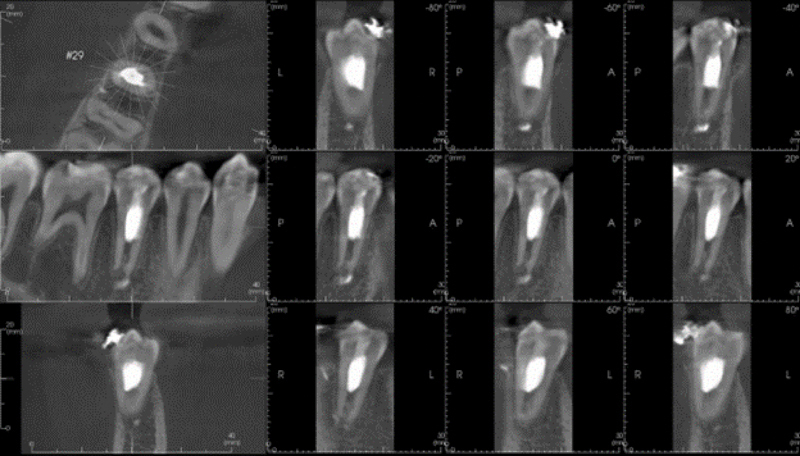
Multiple sagittal and coronal slices of cone beam computed tomography of tooth #29 displaying root endoclosure and no periapical pathology.

**Fig. 15:**
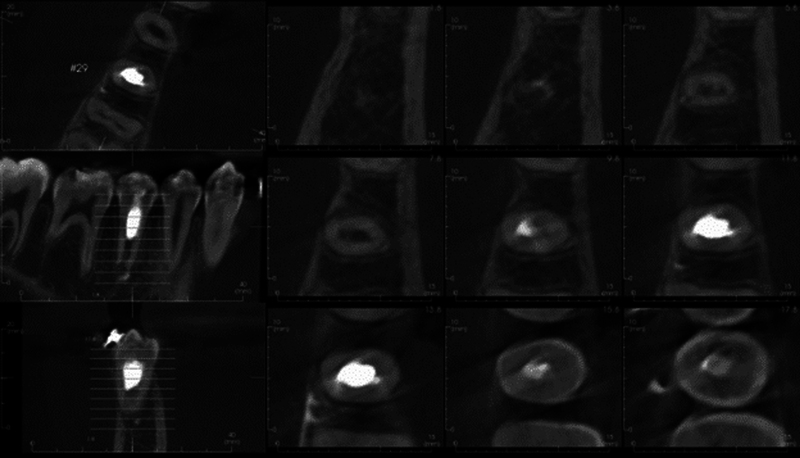
Multiple axial slices of cone beam computed tomography of tooth #29 displaying root endoclosure and no periapical pathology.

The patient returned for a final follow-up appointment 15 months after the initial treatment, where cone beam computed tomography (CBCT) of tooth #29 was obtained. The patient again denied symptoms since the last follow-up appointment, including sensitivity to hot/cold, pain on chewing, spontaneous pain, swelling, and sinus tract. An extraoral examination revealed no significant findings. An intraoral examination of tooth #29 revealed no signs of swelling or sinus tract, no mobility, no isolated deep probing depths, and no percussion (vertical or horizontal) or palpation sensitivity. The occlusal composite restoration remained intact; however, the patient was now in orthodontic braces ( Fig. 13 ). Tooth #29 did not respond to cold or electrical pulp testing at this appointment. The CBCT slices showed no periapical pathology and root end closure ( Figs. 14 and 15 ).

## Discussion

This case displays the successful management of a complex disease progression of a permanent mandibular second premolar in a 10-year-old patient utilizing long-term Ca(OH) _2_ treatment for 6 months and a bioceramic fast set putty as the final obturation material. This particular case was suitable for regenerative treatment because the pulp was necrotic with an open apex with a root less than two-thirds developed. 13 14 However, the highest success rates for regenerative procedures have been found in teeth with apical diameters between 0.5 and 1 mm. 15 Our case's estimated radiographic apical diameter was 1.4 mm at the initial examination visit ( Fig. 2 ), displaying success even though our case to some authors 15 has an apical diameter falling outside of the most desired range. Therefore, the success of our treatment agrees with other evidence suggesting continued root development with apical diameters greater than 1 mm. 16

Long-term Ca(OH) _2_ treatment as an intracanal medicament was not intended in the original treatment plan for this case. It was only intended to use Ca(OH) _2_ for at least 1 week to thoroughly disinfect the root canal and resolve symptoms 17 ; however, due to the relapse of the bacterial infection after the resolution of the acute apical abscess from the initial treatment appointment, a chronic apical abscess formed prior to the second treatment appointment with the presentation of a patent sinus tract. Generally, it is recommended after initial treatment to have the placement of an intracanal medicament for 2 to 4 weeks. 13 In the past, long-term Ca(OH) _2_ application was believed to increase the risk of tooth fracture. However, more recent evidence suggests that the increased risk of fracture depends on the root development stage rather than the use of Ca(OH) _2_ . 18 Due to the severity of this infection and the difficulty in fully resolving it initially, Ca(OH) _2_ had to be replenished after reinstrumentation to a larger size and used for a more extended period of ∼6 months, which may have been essential for the success of this complex case.

It is reported that there is no difference in bacterial reduction capabilities between Ca(OH) _2_ and triple antibiotic paste, the traditional medicament used for regenerative procedures. 19 Furthermore, the detrimental effects of both double (metronidazole and ciprofloxacin) and triple (metronidazole, ciprofloxacin, and minocycline) antibiotic pastes for stem cell viability are not found with Ca(OH) _2_ , 20 and Ca(OH) _2_ promotes stem cell survival and proliferation. 21 The long-term use of Ca(OH) _2_ as an intracanal medicament may have been essential in this case because stem cells from the apical papilla may have been severely disrupted due to the extent and length of apical inflammation. Scaffold and growth factors can be derived from the dentin and fibrin clot, 22 and mesenchymal stem cells can migrate from the apical papilla. 23 The long-term use of Ca(OH) _2_ as an intracanal medicament would have made it essential to preserve any factors or stem cells present in the dentin. Therefore, this case may show an indication for the use of Ca(OH) _2_ as an intracanal medicament in endodontic regeneration cases that display extensive periapical inflammation and destruction because of its protective capabilities for stem cells, even when used for an extended time, in the dentin when other sources may be depleted. These preserved growth factors would then be released by a final rinse with EDTA prior to placement of the obturation material. 24

The successful healing of the periapical tissues within 6 months of completion of treatment and continued root development may be due to the use of EndoSequence BC RRM Fast Set Putty (Brassler). This bioceramic putty has been shown to promote the survival and differentiation of stem cells from the apical papilla. 25 Therefore, the success of using this bioceramic putty may come from utilizing the blood clot created during treatment as a scaffold, which has been shown previously by Bukhari et al to be successful in regenerative treatment. 26 Furthermore, the success of this treatment was dependent on the conservative and quality coronal restoration created by the composite restoration. 27 The conservative access maintained the majority of the coronal tooth structure, which prevented the need for postplacement, which would have prevented the provider from performing the regenerative treatment. It is impossible to perform a regenerative treatment if a core-retaining structure, such as a post, occupies the pulp space. Also, Ricucci et al described that with direct pulp capping, the coronal restoration significantly impacts treatment outcomes. 27 This is especially true for regenerative cases like this one, where it is imperative to avoid reinfection and necrosis of the regenerated tissue. Inflammation can cause tissue destruction, hindering the regenerative process. 28

The success of regenerative endodontics is generally measured by the ability to resolve apical pathosis and increase root length and width. 29 This case displayed the achievement of the primary goal of elimination of symptoms and production of bony healing. 4 It also achieved the secondary goal of increased root wall thickness while achieving minimal to no increase in root length. 4 However, the patient could not achieve the tertiary goal of a positive response to sensibility testing. 4 Therefore, the authors of this case report are satisfied with stating that primary and secondary goals were achieved in this particular case.

## Conclusion

This case report demonstrates that regenerative endodontic treatment can achieve successful results with long-standing, complex periapical infections causing extensive periapical breakdown and bone loss. This case report should help guide providers in treating a unique case like this one with a bioceramic fast set putty with confidence.
